# Supplementary material for: Nicotine aversion is mediated by GABAergic interpeduncular nucleus inputs to laterodorsal tegmentum
Source: Nat Commun. 2018 Jul 13;9:2710. doi: 10.1038/s41467-018-04654-2 (PMC6045623; doi:10.1038/s41467-018-04654-2)
Supplement: Supplementary file 1 — Supplementary Information [file 41467_2018_4654_MOESM1_ESM.pdf]

Supplementary Figures

Nicotine aversion is mediated by GABAergic interpeduncular nucleus inputs to laterodorsal tegmentum

Shannnon L. Wolfman, Daniel F. Gill, Fili Bogdanic, Katie Long, Ream Al-Hasani, Jordan G. McCall, Michael R. Bruchas, and Daniel S. McGehee

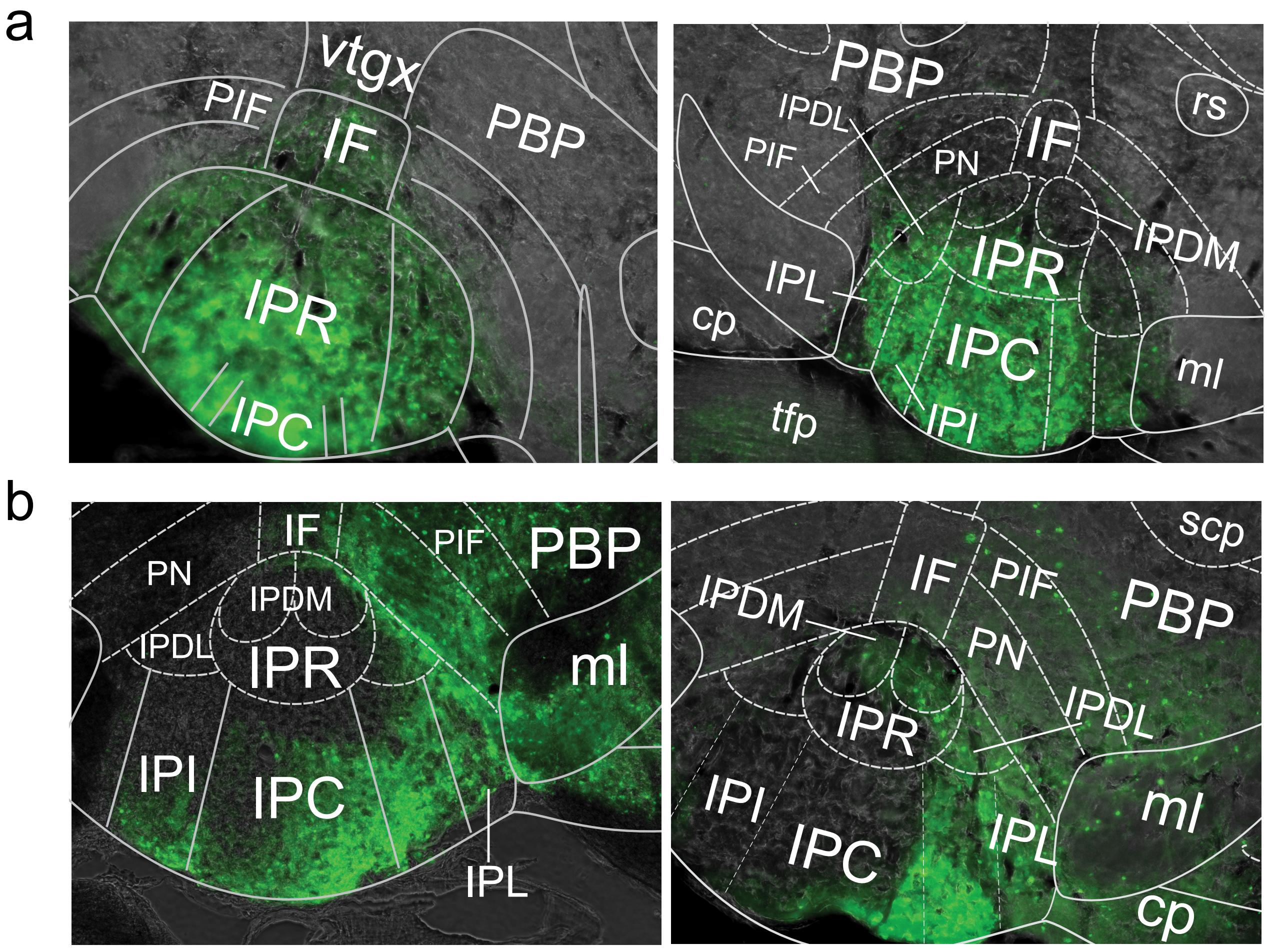

Supplementary Figure 1. Exclusion criteria for IPN viral expression. (**a**) Examples of good expression in the IPN without expression in the VTA. These animals' data were included in analyses. (**b**) Examples of ChR2 expressed substantially in the VTA. These animals' data were excluded from analyses.

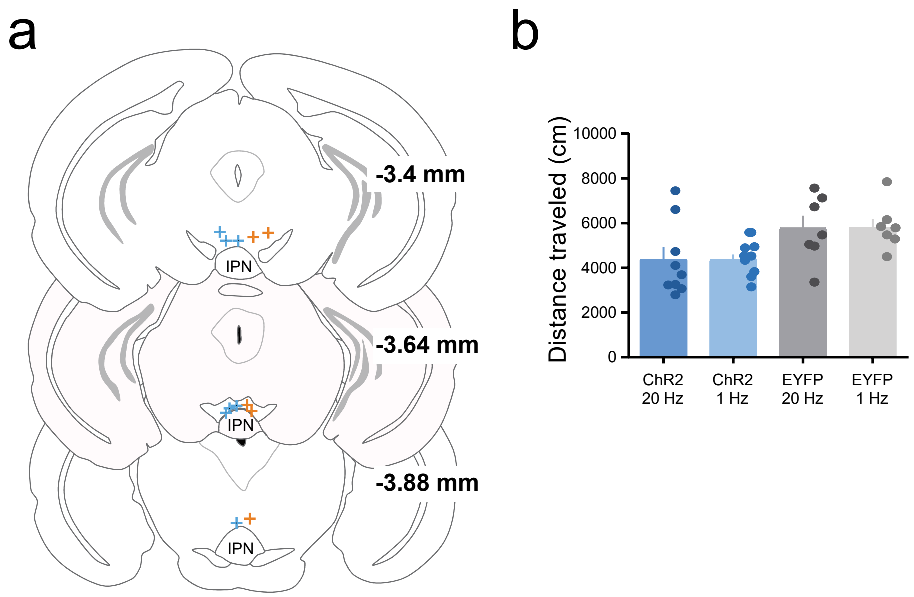

Supplementary Figure 2. Fiber placement and distance traveled during 473 nm light delivery to IPN. (**a**) Fiber optic placements above the IPN (blue-ChR2, orange-EYFP). (**b**) Distance traveled for all groups; one-way ANOVA, $F_{3, 28}=3.234$, $P=0.0372$, Holm-Sidak post-hoc test $P>0.1$ for all individual comparisons. Data are presented as mean ± SEM.

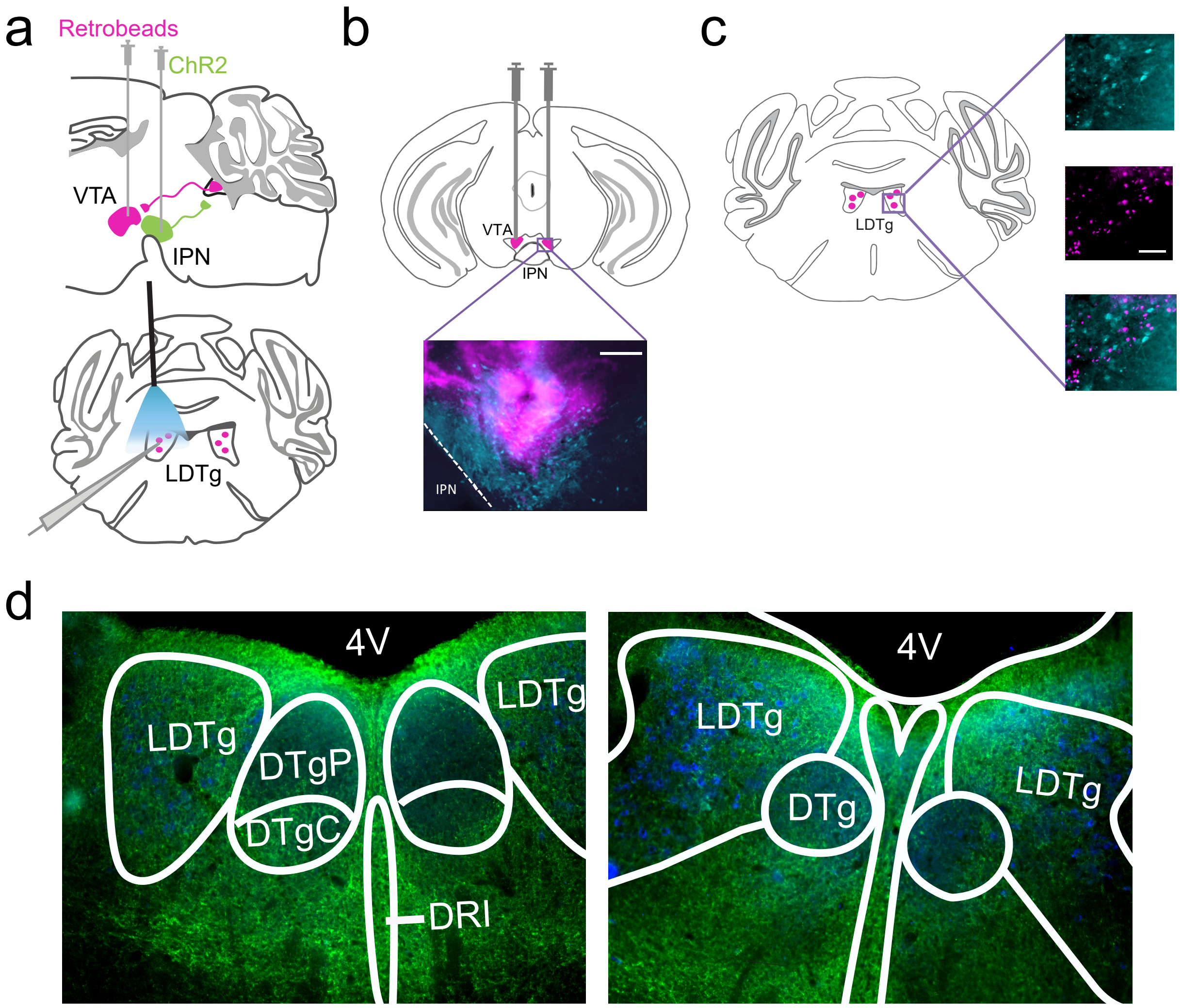

Supplementary Figure 3. Representative images of key experimental components. (**a**) Upper: schematic of surgeries, including retrobead injection into VTA and ChR2 expression in IPN. Lower: recording schematic in which VTA-projecting LDTg neurons are recorded from in slices and blue light is delivered to IPN terminals. (**b**) Upper: schematic of VTA dye injections. Lower: fluorescent image of VTA with retrobeads infused (green-TH, blue-dye). Scale = 200 um (**c**) Left: cartoon demonstrating back-labeled LDTg neurons. Right: fluorescent image of back-labeled LDTg neurons (red) amongst ChAT-positive cells (green) that are characteristic of the LDTg. Scale = 50 um (**d**) Representative images of EYFP-labeled axons from the IPN in the LDTg (green) amongst ChAT-positive cells (blue) that are characteristic of the LDTg.

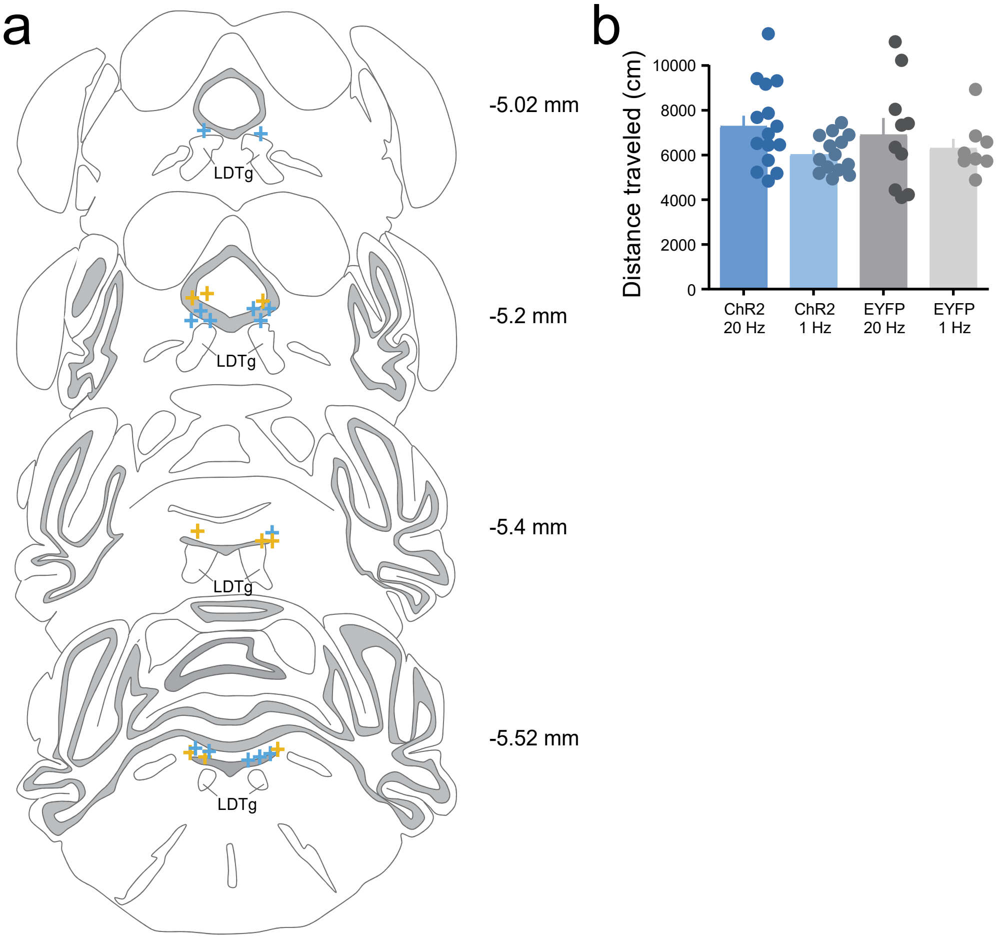

Supplementary Figure 4. Fiber placement and locomotion during 473 nm light delivery to IPN terminals in the LDTg. (**a**) Fiber optic placements above the LDTg (blue-ChR2, orange-EYFP). (**b**) Distance traveled for all groups that received blue light delivery to the LDTg; one-way ANOVA, F3, 43=1.544, P=0.2169, n=15 n=14 n=10 n=8 mice. Data are presented as mean ± SEM.

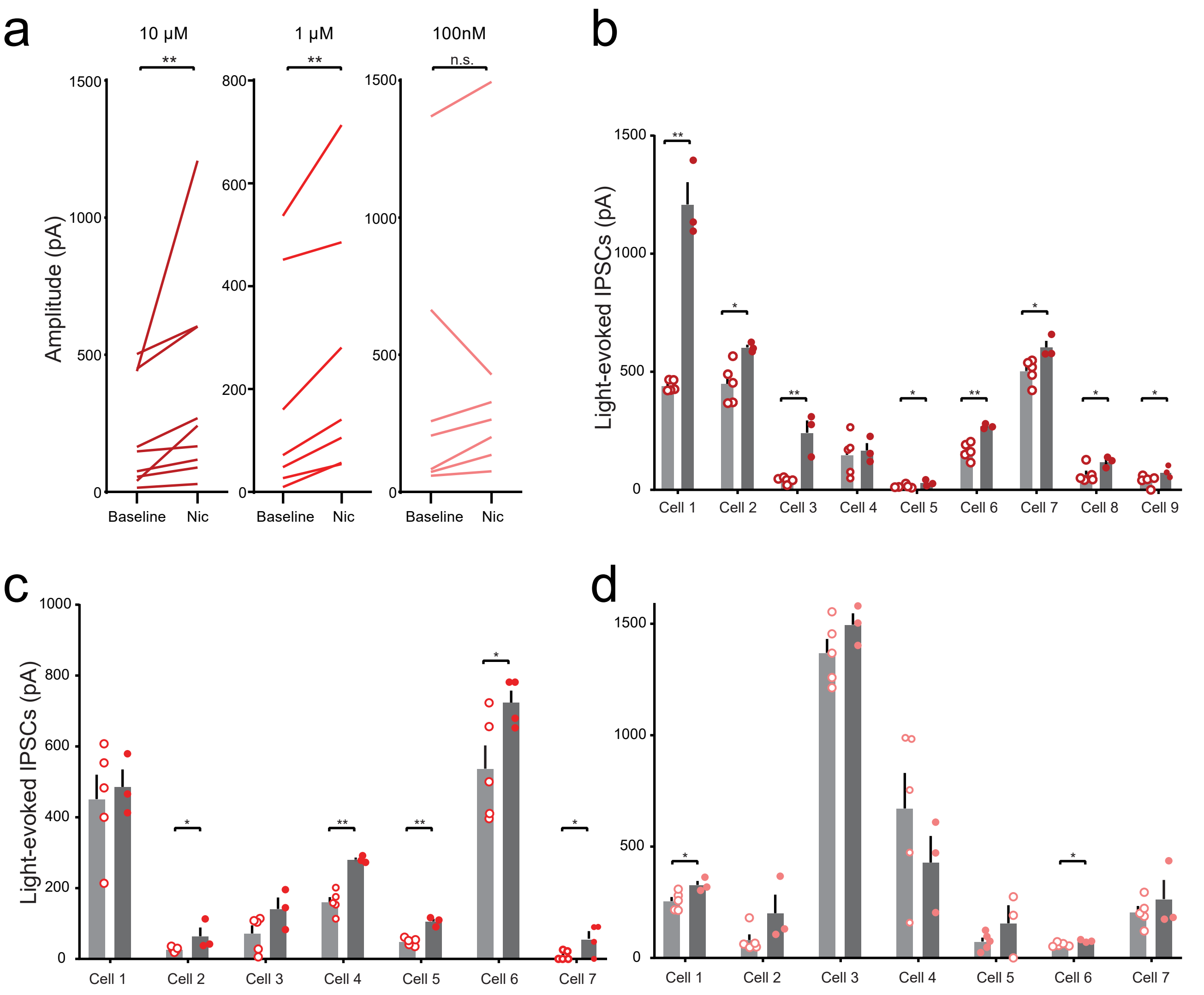

Supplementary Figure 5. Optially evoked IPSC (oIPSC) raw data and within cell comparisons. (**a**) Change in average oIPSC amplitudes recorded from individual LDTg neurons backlabeled from VTA, baseline vs nicotine; ratio paired t-tests (one-tailed): 10μM nicotine, t8=3.492, P=0.0041, n=9; 1μM nicotine, t6=3.367, P=0.0075, n=7; 100nM nicotine, t6=1.708, P=0.0692, n=7 cells. (**b**) Individual oIPSC amplitudes (symbols) and average oIPSC amplitudes (bars, +/- SEM), baseline vs 10μM nicotine; unpaired t-tests (one-tailed): t6=10.93, P<0.0001; t6=3.022, P=0.0117; t6=5.197, P=0.0010; t6=0.3387, P=0.3732; t6=2.095, P=0.0405; t6=4.948, P=0.0013; t6=2.769, P=0.0162; t6=2.271, P=0.0318; t6=2.156, P=0.0372. (**c**) Individual oIPSC amplitudes (symbols) and average oIPSC amplitudes (bars, +/- SEM), baseline vs 1μM nicotine; unpaired t-tests (one-tailed): t6=0.3489, P=0.3695; t6=2.047, P=0.0433; t6=1.788, P=0.0620; t6=6.139, P=0.0004; t6=6.66, P=0.0003; t7=2.33, P=0.0263; t7=2.382, P=0.0244. (**d**) Individual oIPSC amplitudes (symbols) and average oIPSC amplitudes (bars, +/- SEM), baseline vs 100nM nicotine; unpaired t-tests (one-tailed): t6=2.629, P=0.0196; t6=1.735, P=0.0667; t6=1.379, P=0.1086; t6=1.059, P=0.1651; t6=1.281, P=0.1237; t6=2.763, P=0.0164; t6=0.8056, P=0.2256. **P<0.01, *P<0.05.

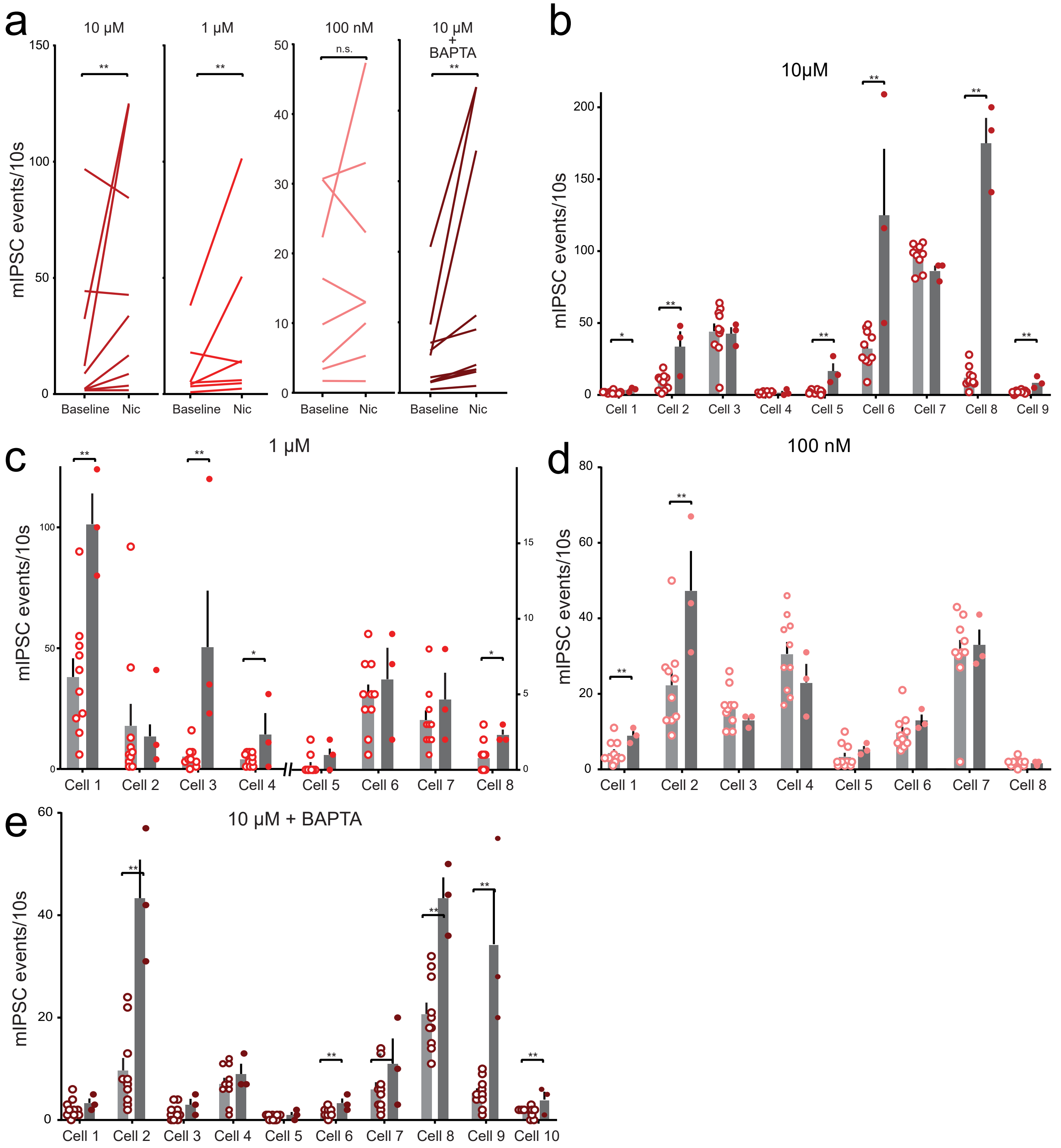

Supplementary Figure 6. mIPSC frequency raw data and within cell comparisons. (**a**) Change in mIPSC frequencies, baseline vs nicotine, for individual LDTg neurons backlabeled from VTA; ratio paired t-tests (one-tailed): 10μM nicotine, t8=3.33, P=0.0052, n=9; t7=3.176, P=0.0078, n=8; t7=1.546, P=0.083; t9=5.479, P=0.0002, n=10 cells. (**b**) Individual mIPSC frequencies (symbols) and within cell averages (bars, +/- SEM), baseline (light bars) vs 10μM nicotine (dark bars); unpaired t-tests (one-tailed): t11=2.685, P=0.0106; t11=4.039, P=0.0010; t11=0.1557, P=0.44; t13=0, P=0.5; t11=5.268, P=0.0001; t12=3.187, P=0.0039; t13=2.732, P=0.086; t13=10.89, P<0.0001; t11=5.026, P=0.0004. (**c**) Individual mIPSC frequencies, baseline vs 1μM nicotine; unpaired t-tests (one-tailed): t11=4.006, P=0.0010; t11=1.428, P=0.0905; t11=3.633, P=0.0020; t11=2.245, P=0.0232; t11=0.632, P=0.2702; t11=0.9304, P=0.1861; t11=0.231, P=0.4108; t11=2.411, P=0.0173. (**d**) Individual raw mIPSC frequencies, baseline vs 100nM nicotine; unpaired t-tests (one-tailed): t12=3.178, P=0.0040; t11=2.869, P=0.0076; t12=1.193, P=0.1280; t12=1.197, P=0.1272; t12=1.535, P=0.0754; t11=1.108, P=0.1457; t11=0.3285, P=0.3743; t11=0.05118, P=0.4800. (**e**) Individual raw mIPSC frequencies, BAPTA+baseline vs BAPTA+10μM nicotine; unpaired t-tests (one-tailed): t11=1.005, P=0.1682; t11=5.743, P<0.0001; t11=1.416, P=0.0923; t11=0.7953, P=0.2216; t11=1.188, P=0.1300; t11=2.764, P=0.0092; t19=1.743, P=0.0487; t13=5.127, P<0.0001; t12=5.671, P<0.0001; t14=3.424, P=0.0021. **P<0.01, *P<0.05.

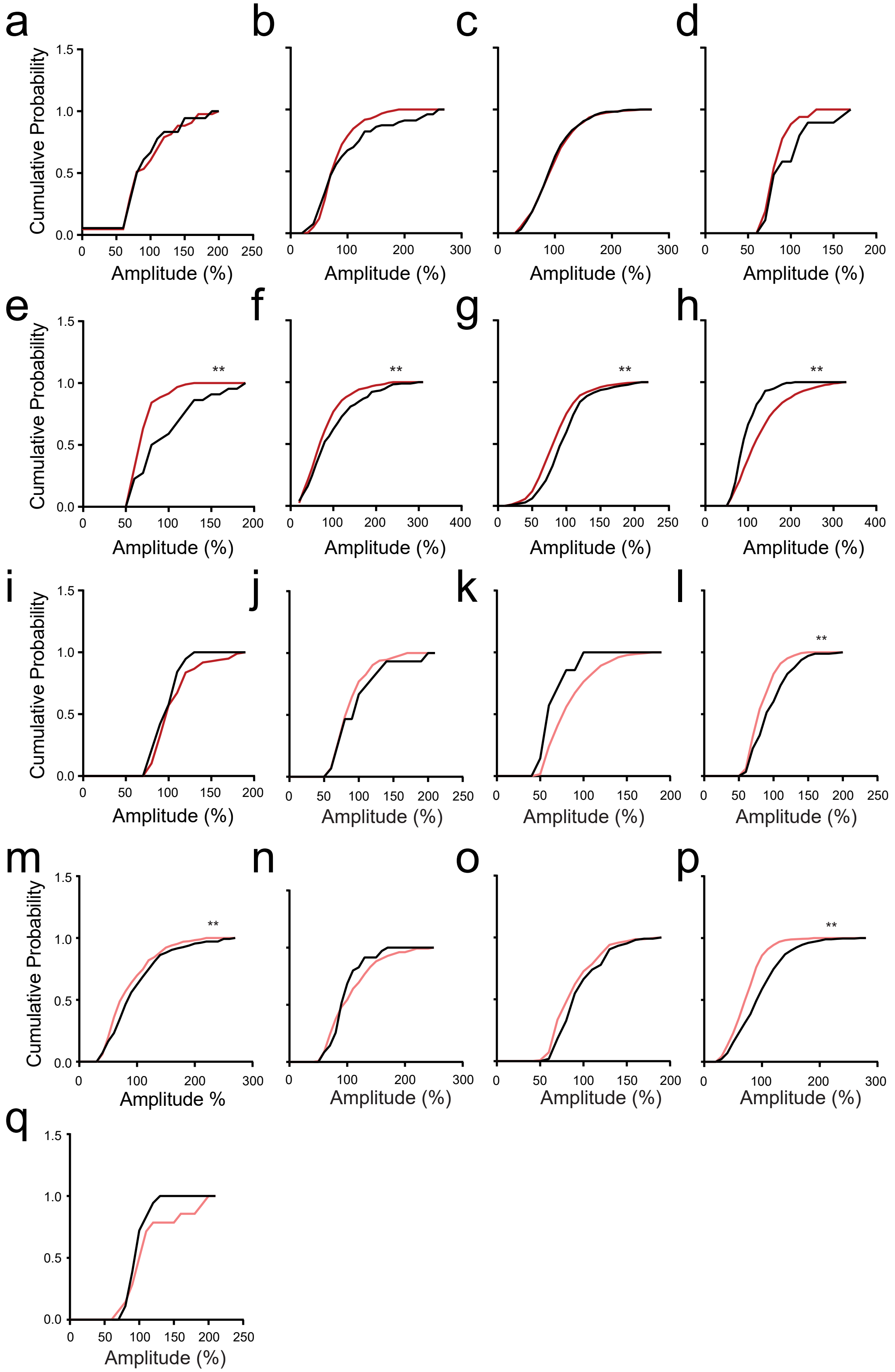

Supplementary Figure 7. mIPSC raw amplitudes. (**a-i**) Cumulative probability amplitude histograms, baseline (black) vs 10μM nicotine (red). Kolmogorov-Smirnov tests: D=0.1189, P=0.9939; D=0.1765, P=0.0534; D=0.05961, P=0.1772; D=0.3622, P=0.1897; D=0.4292, P=0.0028; D=0.1462, P<0.0001; D=0.1829, P<0.0001; D=0.2908, P<0.0001; D=0.2229, P=0.4079. (**j-q**) Cumulative probability amplitude histograms, baseline (black) vs 100nM nicotine (red). Kolmogorov-Smirnov tests: D=0.1839, p=0.7406; D=0.4232, p=0.1676; D=0.2375, p=0.0001*; D=0.1553, p=0.0002*; D=0.1863, p=0.2988; D=0.1534, p=0.0662; D=0.2829, p<0.0001*; D=0.2381, p=0.7634.

** P<0.01 Note that although nicotine induced IPSC amplitude changes in some recordings, most were decreases. Overall the data support the idea that the nicotine-induced change in mIPSC frequency occurs via presynaptic nAChR activation.

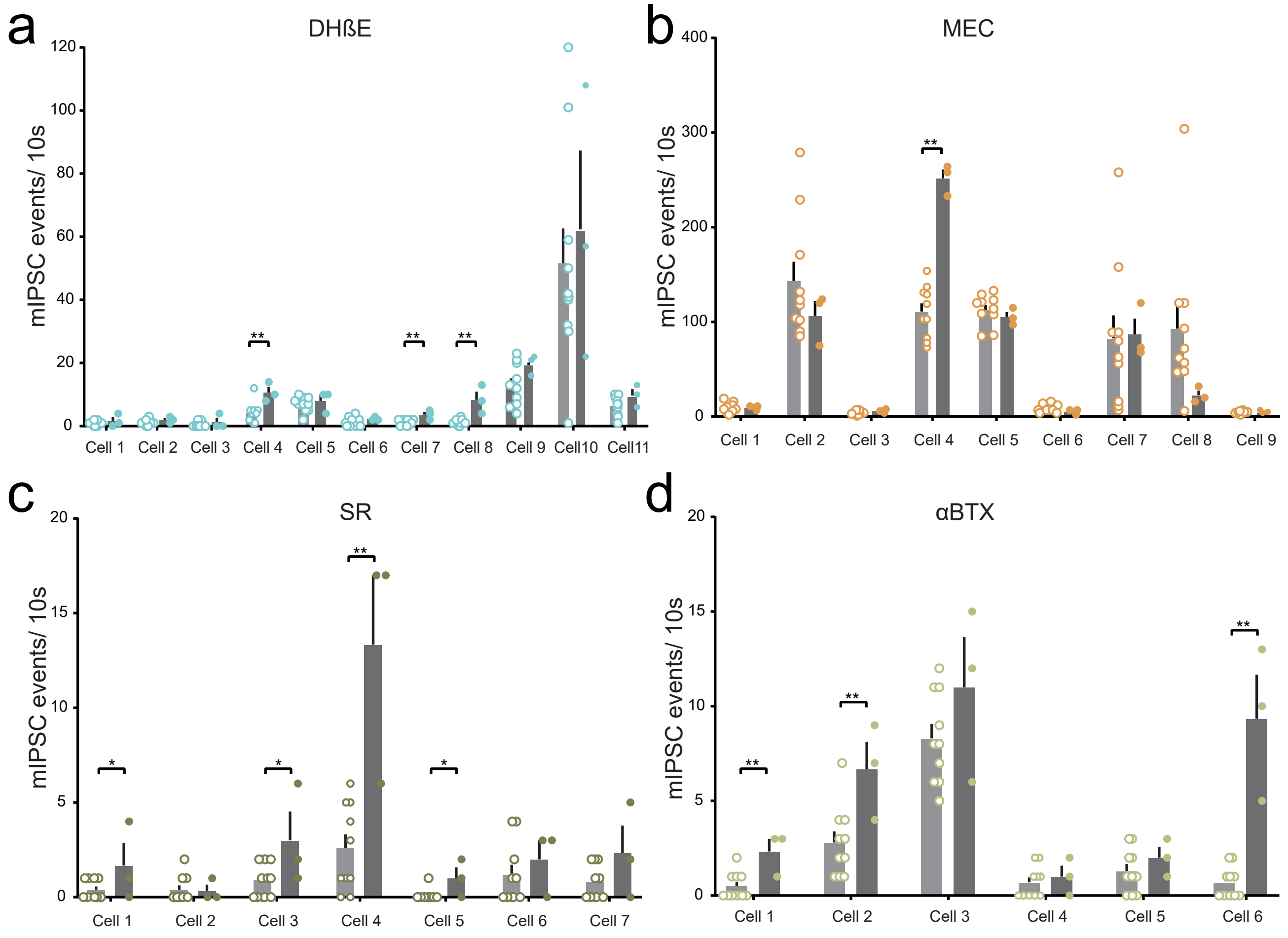

Supplementary Figure 8. Within cell pharmacological assessment of nAChR subtype contribution to nicotine-induced enhancement of mIPSC frequency. (**a**) Individual raw mIPSC frequencies (symbols) and averages (bars, +/- SEM), DHβE+baseline vs DHβE+10μM nicotine; unpaired t-tests (one-tailed): $t11=1.156$, $p=0.1361$; $t11=1.593$, $p=0.0697$; $t13=1.408$, $p=0.0913$; $t11=3.104$, $p=0.0050$; $t11=0.6404$, $p=0.2675$; $t11=1.268$, $p=0.1154$; $t12=4.002$, $p=0.0009$; $t11=5.074$, $p=0.0002$; $t11=1.638$, $p=0.0649$; $t11=0.4465$, $p=0.3319$; $t11=1.541$, $p=0.0758$. (**b**) Individual raw mIPSC frequencies (symbols) and averages (bars, +/- SEM), MEC+baseline vs MEC+10μM nicotine; unpaired t-tests (one-tailed): $t11=0.231$, $P=0.4108$; $t11=0.947$, $P=0.1820$; $t11=1.398$, $P=0.0949$; $t11=8.296$, $P<0.0001$; $t11=0.7394$, $P=0.2376$; $t11=1.131$, $P=0.1411$; $t11=0.09276$, $P=0.4639$; $t11=1.438$, $P=0.0891$; $t11=0.9392$, $P=0.1839$. (**c**) Individual raw mIPSC frequencies (symbols) and averages (bars, +/- SEM), SR+baseline vs SR+10μM nicotine; unpaired t-tests (one-tailed): $t11=1.918$, $P=0.0407$; $t11=0.1492$, $P=0.4420$; $t11=2.314$, $P=0.0205$; $t11=4.797$, $P=0.0003$; $t11=2.663$, $P=0.0110$; $t11=0.7408$, $P=0.2372$; $t11=1.716$, $P=0.0571$. (**d**) Individual raw mIPSC frequencies (symbols) and averages (bars, +/- SEM), αBTX+baseline vs αBTX+10μM nicotine; unpaired t-tests (one-tailed): $t11=3.45$, $P=0.0027$; $t11=2.928$, $P=0.0069$; $t11=1.402$, $P=0.0942$; $t11=0.5311$, $P=0.3030$; $t11=0.9392$, $P=0.1839$; $t11=6.986$, $P<0.0001$. $^{**}P<0.01$, $^{*}P<0.05$.

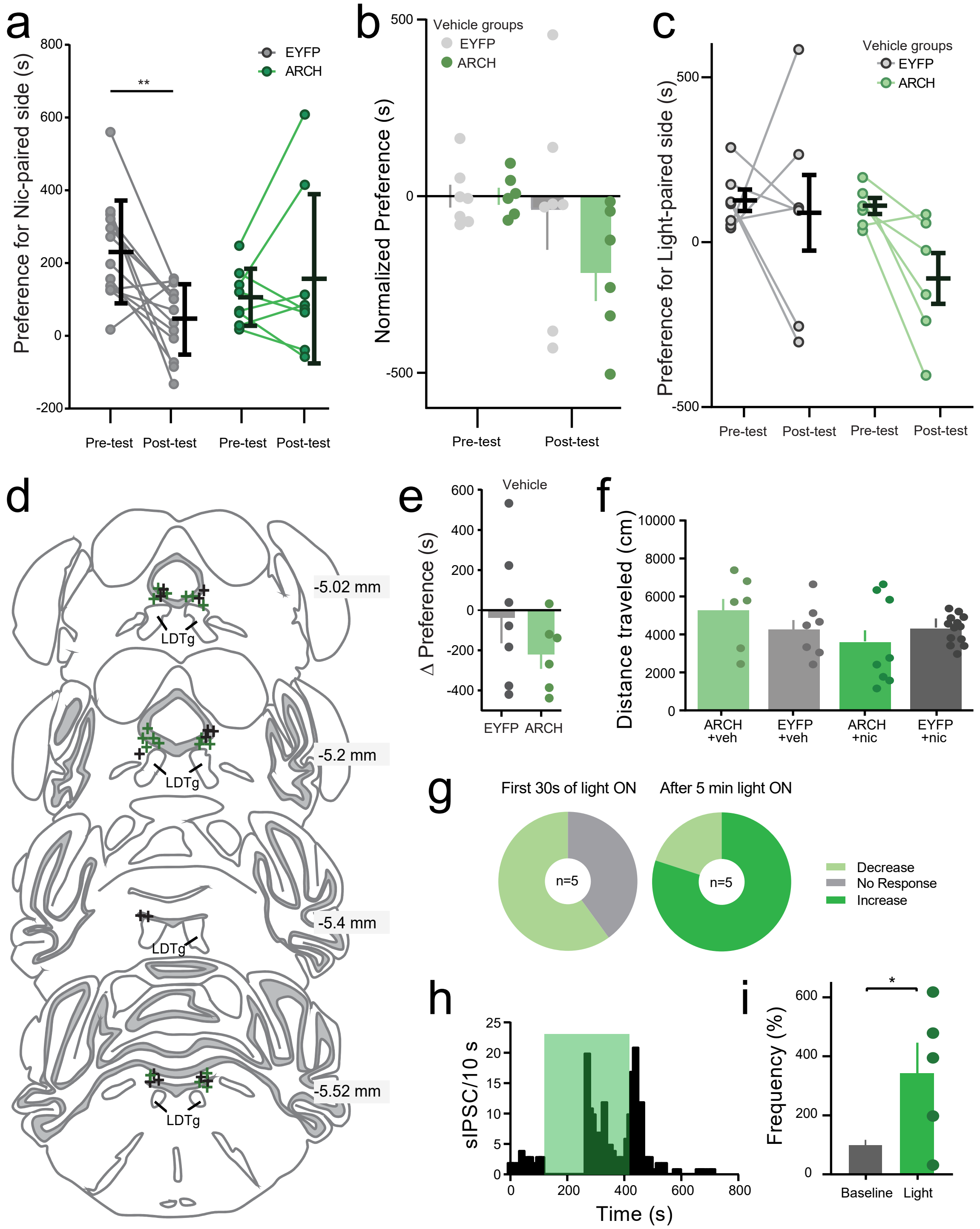

Supplementary Figure 9. ARCH behavioral controls and physiology. (**a**) Raw preference scores for nicotine-treated groups; 2-way RM ANOVA (Time: F1,19=2.975, P=0.1008; Group: F1,19=0.01494, P=0.9040; Interaction: F1,19=9.109, P=0.0071); Bonferroni post-hoc (EYFP pre vs post: t19=3.842, P=0.0022, n=13), (ARCH pre vs post: t19=0.8218, P=0.8428, n=8). (**b**) Normalized preference scores for EYFP (gray) and ARCH (green) mice before and after 3 days of conditioning during which green light was delivered in the LDTg without being paired with nicotine; 2-way RM ANOVA (Time: F1, 11=2.812, P=0.1217; Group: F1, 11=2.254, P=0.1614; Interaction: F1, 11=1.402, P=0.2613); Bonferroni post-hoc (EYFP pre vs post: t11=0.3625, P>0.9999, n=7), (ARCH pre vs post: t11=1.949, P=0.1544, n=6). (**c**) Raw preference scores for vehicle control groups. (**d**) Fiber optic placements above the LDTg for ARCH CPA experiments. (**e**) Change in preference for initially preferred side after vehicle conditioning; unpaired t-test (two-tailed), t11=1.184, P=0.2613. (**f**) Distance traveled for all ARCH CPA conditions; one-way ANOVA, F3, 30=0.4599, P=0.7123, n=13, n=8, n=6, n=7 mice. (**g**) Pie charts showing cell sIPSC responses to light delivery at different time points. (**h**) Example sIPSC frequency histogram showing the time course of sIPSC changes during light exposure. (**i**) sIPSC frequency, baseline vs after 5 min of light; paired t-test (one-tailed), t4=2.342, P=0.0396, n=5 from 3 cells, 2 mice. Data are presented as mean +/- SEM. **P<0.01, *P<0.05.
